# Ecdysone Receptor (EcR) and Ultraspiracle Protein (USP) Genes From *Conopomorpha sinensis* Bradley Eggs: Identification and Expression in Response to Insecticides

**DOI:** 10.3389/fphys.2020.00851

**Published:** 2020-07-17

**Authors:** Qiong Yao, Shu Xu, Yizhi Dong, Linfa Quan, Bingxu Chen

**Affiliations:** ^1^Guangdong Provincial Key Laboratory of New High Technology for Plant Protection, Plant Protection Research Institute, Guangdong Academy of Agricultural Sciences, Guangzhou, China; ^2^Guangdong Laboratory for Lingnan Modern Agriculture, Guangzhou, China

**Keywords:** litchi fruit borer, EcR, USP, ovicide, biomarker

## Abstract

*Conopomorpha sinensis* Bradley (Lepidoptera: Gracilariidae) is the dominant insect pest of litchi (*chinensis* Sonn.) and longan (*Euphoria longan* Lour.) fruit trees. Management of this pest species is a challenging task due to its cryptic borer behavior. Controlling *C. sinensis* at the egg stage is the best alternative strategy to chemical control of *C. sinensis* adults. However, thorough studies regarding the indirect and sublethal effects of chemicals on the different developmental stages of *C. sinensis* are insufficient. In this study, the effect of some insecticides was evaluated on *C. sinensis* eggs. The ovicidal activity of chlorbenzuron, abamectin, chlorantraniliprole, and λ-cyhalothrin was confirmed by morphological observation of the defects in *C. sinensis* eggs. Moreover, we characterized four essential ecdysone receptor proteins in insects [i.e., two isoform ecdysone receptors (EcR: CsEcRA. CsEcRB) and two isoform ultraspiracle proteins (USP: *Cs*USP1, *Cs*USP2)] from *C. sinensis* eggs. The *CsEcRA*, *CsEcRB*, *CsUSP1*, and *CsUSP2* genes consisted of 1521-, 1614-, 1410-, and 1236-bp open reading frames which encoded proteins of 506, 527, 469, and 413 amino acid residues, respectively. Furthermore, the embryonic differential responses of *CsEcR*s, *CsUSP*s, and vitellogenin receptor (*VgR: CsVgR*) to insecticides were evaluated by qRT-PCR. Among the five tested genes, *CsVgR* and *CsUSP1* were the most sensitive to all the tested insecticides, with fold change of the expression diminished by 4.27–8.70 times compared with untreated control insects. The data suggests that these insecticidal compounds regulate the expression of these specific proteins, which might eventually lead to reduced viability of *C. sinensis* eggs. We present here the first data providing molecular elucidation of ecdysone receptor genes and their differential responses to insecticides in *C. sinensis* eggs. Together with our previous report of insecticide sublethal effects on two reproduction-related genes in *C. sinensis* adults, *CsVgR* and *CsUSP1* seem to be appropriate molecular parameters for the evaluation of insecticide impact on *C. sinensis*. This study exemplifies the potential utility of transcriptional measurement of nuclear receptors as the molecular biomarkers for ecotoxicological evaluations of ovicidal impact of insecticides.

## Introduction

Litchi (*chinensis* Sonn.) and longan (*Euphoria longan* Lour.) (both Sapindaceae) are two dominant and valuable fruits in southeast Asia and southern China. The cultivated area and fruit yield of these two fruits in China are the highest in the world (Chen et al., 2013). *Conopomorpha sinensis* Bradley (Lepidoptera: Gracilariidae), also referred to as the litchi fruit borer, is the most destructive Lepidoptera pest of litchi and longan (Li et al., 2018). *C. sinensis* is considered a great threat to the tropical fruit industry due to the severe economic damage that it causes in litchi and longan production in China. Management of this pest species is a big challenge due to its cryptic borer behavior. *C. sinensis* females lay an average of 13.2 eggs per day during its oviposition period (Yao et al., 2019). After hatching, the *C. sinensis* larvae burrow directly into the fruit, where they feed until pupation. It is difficult to detect or control *C. sinensis* following egg hatching since the complete larval development occurs inside the host plant.

Insecticides are crucial tools aimed at reducing the pest population density and minimizing economic losses caused by pests. In China, there are only 8 pesticide products officially registered with validity for *C. sinensis* control in 2019 according to the China Pesticide Information Network. Specifically, they are pyrethroids (including *λ*-cyhalothrins, alphamethrin, and cypermethrin), chlorpyrifos, diflubenzuron, and mixtures of chlorpyrifos and triazophos together with pyrethroid (Supplementary Table S1). The effectiveness of insecticides mainly depends on the timing of their application in response to the pest developmental stages (Guedes et al., 2016; Nozad-Bonab et al., 2017). The vast majority of pesticide products mentioned above are used to control *C. sinensis* adult. One way to improve the control efficiency is to target other developmental stages of *C. sinensis*. Hence, controlling *C. sinensis* at the egg stage may assist in its population management. However, eggs are relatively difficult targets for insecticide application because of their protective structure and sessile condition at concealed sites (Smith and Salkeld, 1966; Smagghe et al., 2019).

Under hormonal control, insects undergo extensive tissue proliferation and morphogenesis during embryogenesis (Baehrecke et al., 1993; Dong et al., 2003). The involvement of the ecdysone signaling pathway in the embryonic development of insects has been elaborately studied in *Drosophila melanogaster* and some other insects (D’avino and Thummel, 2000; Maestro et al., 2005; Nowickyj et al., 2008). In the target tissue, ecdysone is converted into its active form (20E). After binding to a dimer formed by ecdysone receptor (EcR) and ultraspiracle protein (USP), the 20E-EcR-USP complex subsequently regulates numerous targets including a conserved transcription factor network, which is also referred to as the “Ashburner cascade” (Smith and Salkeld, 1966; Truman, 2019). The expression of ecdysone receptor (*EcR*) has been correlated with the initiation of embryonic morphogenesis in *Copidosoma floridanum* (Hymenoptera: Encyrtidae) (Baehrecke et al., 1993). The requirement of EcR for hatching was also confirmed in a conditional rescue system in *D. melanogaster* (Li and Bender, 2000). The involvement of EcR-B1 and USP in expression of eggshell gene *VM32E* was confirmed during *Drosophila* oogenesis (Bernardi et al., 2009). In the sexual differentiation of the *Bombyx mori* embryo, the double sex gene was regulated by *EcR-A* and early late gene *E75* (Matsushima et al., 2019). Obviously, the ecdysone signaling pathway plays an essential role in the embryonic development of insects. The functions of the steroid hormone ecdysone during embryonic metamorphosis have been well established, but the roles of the embryonic response of the ecdysone signaling pathway to hazardous chemicals (such as insecticides) remain poorly understood.

Insecticides may interfere with different physiological and biochemical processes as well as numerous behaviors in insects (Guedes et al., 2016). Additionally, a cascade of changes in the expression of regulatory factors is sequentially upregulated or downregulated through topical, residual, or dietary exposure to insecticides at different developmental stages of insects (Nakagawa, 2005; Park and Kwak, 2018). However, contrasting with the majority of efforts focused on the adult and larval stages of target pest species, research into the toxic effects of such compounds on other developmental stages of insects has been neglected (Smagghe et al., 2019). Eggs of insect pests are difficult targets for insecticide application despite being perceived as the most vulnerable stage. Furthermore, no previous studies have been conducted examining the ovicidal effects of insecticides in *C. sinensis*. The purpose of this study was to investigate the ovicidal effects of four environmentally friendly and most commonly used insecticides on *C. sinensis* eggs. In addition, we examined embryonic expression variation of ecdysone receptors (*EcR* and *USP*) and the vitellogenin receptor (*VgR*) after exposure to insecticides in *C. sinensis*. This study provides broader insight into the molecular responses underlying the ovicidal effects of insecticides on target pests as well as exploring the potential utility of ecdysone receptor transcriptional measurement as a rapid biomarker for impact evaluation of environment agents.

## Materials and Methods

### Insect Rearing and Collection

*C. sinensis* pupae were collected from fallen fruits as previously described (Yao et al., 2019). *C. sinensis* adults were raised in incubators (26 ± 1°C), 65–85% RH, with a 14:10 h (L:D) photoperiod ratio and 20% (v/v) diluted honey as a food source. Fresh litchi fruits were collected in a litchi orchard, were washed thoroughly with distilled water, and were used as oviposition stimulants. The eggs were collected daily and kept in incubators for later use.

### RNA Extraction and cDNA Synthesis

Total RNA was extracted from 300 *C. sinensis* eggs (including 100 1-, 2-, 3-day-old eggs, respectively) using Trizol reagent (Invitrogen, Boston, MA, United States). The RNA sample was dissolved in diethylpyrocarbonate (DEPC)-treated H_2_O (Tiangen, Beijin, China), and the RNA integrity was confirmed using agarose gel electrophoresis. First-strand cDNA was synthesized from 1 μg of total RNA in a 20 μl reaction mixture using a PrimeScript RT Reagent Kit with gDNA Eraser (Takara, Tokyo, Japan).

### Identification of Ecdysone Receptors (*CsEcRs*) and Ultraspiracle Proteins (*CsUSPs*) in *C. sinensis* Eggs

Four pairs of degenerate primers (EcRcom-F1/EcRcom-R1, EcRcom-F2/EcRcom-R2, USPcom-F1/USPcom-R1, and USPcom-F2/USPcom-R2) (Table 1) were designed on the basis of the conserved region of other insect EcR and USP cDNAs. PCR was performed to obtain partial cDNA sequences using TransTaq DNA Polymerase High Fidelity (Transgene Biotech, Beijing, China). PCR conditions were as follows: 94°C for 5 min; five cycles of 94°C for 40 s, 48°C for 1 min and 72°C for 40 s; 25 cycles of 94°C for 40 s, 53°C for 1 min and 72°C for 40 s; a final extension at 72°C for 6 min. The amplified products were purified using Gel Extraction Kit (Tiangen, Beijin, China). The purified PCR products were subcloned into the pMD 18-T vector (TakaRa, Tokyo, Japan) and transformed into *Escherichia coli* DH5α-competent cells (Tiangen, Beijing, China). Positive clones were confirmed by PCR and automated sequencing [The Beijing Genomics Institute (BGI) China].

**TABLE 1:** Primers used in this study.

**For EcRs or USPs of *C. sinensis* cloning**			
**Degenerate primers (5′–3′)**			
EcRcom-F1		TGTGAAGGDTGYAAAGGWTTC	
EcRcom-R1		GTCATYTCBGTGATYTGDCGGA	
EcRcom-F2		TCGCVAGGCTVMTSTGGTACCA	
EcRcom-R2		GGTAGTAYCKCTGGATCTC	
USPcom-F1		AACTAYCCBCCNAAYCAYCC	
USPcom-R1		ATYTGRCARAGRSWGGAGAC	
USPcom-F2		GARATGGARKCNCTGGTBGCDGA	
USPcom-R2		TCGAAGCTCTTKAGBGAKATGGA	
**Nested gene-specific primers (5′-3′)**			
5′-EcR-R1		ACACGCATGTCCGAACTTAC	
5′-EcR-R2		TCATTCCTACCGCTAGACA	
5′-USP-R1		CTTCTGGTATCGACAGTACTGA	
5′-USP-R2		TGCTTTCCAGATGCCCTATCACCG	
3′-EcR-F		CGTCCAGCGAGGTGATGATGC	
3′-USP-F		GACCAGGCCGAGTACGTCGCGCT	
**For quantitative real-time RT-PCR**			

**Gene**	**Forward (5′–3′)**		**Reverse (5′–3′)**

EcRA	AGACGAACAATGGCTACTC		CACCCGTTTACACTGGAC
EcRB	TTCCACACTCTGCGAATGC		ACATGCCGTCGTCGTAGCC
USP1	ACCGTGGCGAAGAAAGATAAG		ATTGCATGTCGAGAGAACAGTC
USP2	GAACCCTCTCGAGATCCAG		AATTGAGTTGGGTGAGGTGG
β-Actin	AGATCTGGCACCACACCT		ACGATACCGGTGGTACGAC

Full-length sequences of *CsEcR*s and *CsUSP*s were obtained using the SMART RACE (rapid amplification of cDNA ends) cDNA Amplification kit (Clontech, Mountain View, CA, United States). The nested gene-specific primers for *CsEcR*s and *CsUSP*s (Table 1) were designed based on the partial cDNA sequence obtained as described above. The 5′-RACE and 3′-RACE were performed using gene-specific primers and universal anchor primers (Universal Primer Mix/UPM and Nested Universal Primer A/NUP, Clontech). The RACE products were separated by agarose gel electrophoresis, purified, sub-cloned into vectors using pGEM-T Easy vector (Promega, Madison, WI, United States), and sequenced as described above. The overlapping sequences of the PCR fragments were assembled to obtain the full-length *CsEcR* and *CsUSP* cDNAs. Each kit was used according to the manufacturer’s instructions.

### Characterization of *CsEcRs* and *CsUSPs*

The sequences of *C. sinensis EcR* and *USP* cDNAs were compared with those of other *EcR* and *USP* sequences in GenBank using the “BLAST-N” or “BLAST-X” tools available from NCBI. The open reading frames of the *CsEcR* and *CsUSP* genes were obtained using ORF finder^1^. The amino acid sequences of *CsEcR*s and *CsUSP*s were deduced from the corresponding cDNA sequences using the translation tool on the ExPASy Proteomics website^2^. Various physical and chemical parameters for the CsEcRs and CsUSPs proteins were performed with analysis tools from the ExPASy ProtParam tool^3^. The transmembrane helices were analyzed by TMHMM Server v.2.0^4^. The signal peptide cleavage site was predicted using the SignalP 4.1 Server^5^. Cellular localization was predicted by PSORT II^6^. The NCBI Conserved Domain Database (CDD^7^) was used to analyze conserved domains.

The sequence of the *CsEcR* and *CsUSP* cDNAs were individually compared with other available Lepidoptera *EcR* and *USP* sequences deposited in GenBank using the BLAST-X tool on the NCBI website. Multiple sequence alignments of the deduced CsEcR and CsUSP amino acid sequences were made using Clustalw^8^. Phylogenic and evolutionary analyses were conducted by Molecular Evolutionary Genetics Analysis (MEGA) software v.5.05 using a neighbor-joining (NJ) method with bootstrap of 1,000 replicates.

### Effects of Insecticides on *C. sinensis* Eggs

To evaluate the activity of insecticides, we performed a mortality bioassay using 1-, 2-, and 3-day-old *C. sinensis* eggs. Preliminary experiments were conducted to assess the concentration ranges of the insecticides. The eggs were submerged for 5 s in tested concentrations of chlorpyrifos, λ-cyhalothrin, chlorbenzuron, and tebufenozide (Hepeng, Shanghai, China, and Altas Scientific, Tianjin, China) diluted with Tween-80. The ranges of concentrations were presented in Supplementary Table S2. In the control group, eggs were immersed for 15 s in distilled water with Tween-80. Eggs from each treatment were kept separate for hatching assessment. All the eggs were microscopically examined daily, and the hatched eggs were counted until no eggs hatched for at least 48 h (Dong et al., 2015). All the bioassays were simultaneously carried out under the same conditions. All insecticides were examined using five concentrations with 40 individuals were per replicate, three replicates per dose, and five doses per assay. The mortality for the treated group was corrected for natural mortality in the control group using Abbott’s formula when the control mortality is ≥ 5%. To generate a concentration-mortality regression line for each chemical, all data were subjected to Probit analysis using PROC PROBIT (SAS Institute, 2008).

### Quantitative Real-Time PCR (qRT-PCR) Analysis

qRT-PCR analysis was performed to determine the developmental expression pattern of four *EcR* and *USP* genes, and the expression of selected genes in 1-day-old *C. sinensis* eggs after exposure to sublethal doses of tested insecticides. Total RNA isolation and cDNA synthesis followed the processes described above. Three individual samples were prepared from 200 eggs selected randomly from the treatment and control groups at 24 h after exposure. The cDNAs were diluted 1:10 with distilled water equivalent to 5 ng of total RNA and were used as templates in qRT-PCR analysis. The mRNA transcripts of *CsEcR*, *CsUSP*, and *CsVgR* were assessed using the GoTaq qPCR Master Mix (Promega, Madison, WI, United States) with the specific primers described in Table 1. The PCR conditions were as follows: hot-start activation at 95°C for 2 min, 40 cycles of denaturation at 95°C for 15 s and extension at 58°C for 1 min, followed by a final dissociation at 72°C. The *C. sinensisβ-Actin* (GenBank accession NO. KF598848) was chosen as a suitable internal control gene, and the negative controls, containing no cDNA template, were included in the experiments. The melting curve analysis and standard curve analysis were performed to check the homogeneity of the PCR product and efficiency of primers. The relative gene expression levels in developmental expression pattern analysis were calculated according to 2^–ΔΔCt^ method, whereas the fold changes due to treatments were calculated according to the comparative Ct method (Schmittgen and Livak, 2008).

### Statistical Analysis

The LC_50_ ratio for each insecticide was tested for significance according to Robertson and Priesler (1992) to determine differences at *P* > 0.05, which was achieved by calculating the corresponding 95% confidence intervals (Robertson and Priesler, 1992). Values were expressed as mean ± *SD*. Statistical analyses were performed by one-way analysis of variance (ANOVA) followed by Tukey’s test for multiple comparisons in SPSS 18.0. Differences were considered significant at *P* < 0.05 (SAS Institute, 2008).

## Results

### Cloning of CsEcRs and CsUSPs

In order to obtain sequences encoding *C. sinensis* EcRs, a variety of degenerate oligonucleotide primers were designed complementary to the most highly conserved coding sequences of nuclear receptor superfamily members from other lepidopteran insects (Table 1). Two fragments of 744 and 927 bp were obtained. Then we subsequently conducted 5’-RACE and 3’-RACE using nested gene-specific primers, and two cDNA sequences were isolated after combining fragments. A database search was conducted using the BLAST program, and the two deduced amino acid sequences were found to have structures typical for the nuclear receptor superfamily and were found to be highly homologous to previously reported EcR A- and B-isoforms of other insects. The sequenced *CsEcRA* gene encoding an open reading frame (ORF) is 1521 bp in length and corresponds to a predicted protein with 506 amino acids (56.59 KDa) (Table 2). The ORF of *CsEcRB* is 1614 bp in length and corresponds to a predicted protein with 537 amino acids (60.44 KDa).

**TABLE 2:** Details of *C. sinensis* ecdysone receptor (EcR) and ultraspiracle protein (USP) sequences.

**Gene**	**Accession number**	**ORF (kb)**	**Protein (AAs)**	**MW (KDa)**	**pI**	**Cellular localization**	**Highest AA composition**	**GRAVY**	**Aliphatic index**	**Instability**	**SP (AAs)**
EcRA	KY025550	1,521	506	56.59	7.79	Nuclear (56.5%)	Leu (L)9.3%	-0.392	81.54	Unstable	None
EcRB	MT022457	1,614	537	60.44	6.25	Mitochondrial (52.2%)	Leu (L) 10.2%	-0.416	77.91	Unstable	None
USP1	MT022458	1,410	469	52.65	8.27	Nuclear (60.9%)	Leu (L) 11.5%	-0.374	81.17	Unstable	None
USP2	MT022459	1,236	413	46.79	7.87	Nuclear (82.6%)	Leu (L) 11.9%	-0.423	83.41	Unstable	None

**AA, Amino acid; MW, Molecular weight; GRAVY, Grand average of hydropathicity; pI, isoionic point; SP, signal peptiede.**

In a similar manner, we cloned the cDNA sequence of USP from *C. sinensis* eggs. In the process of cloning and sequencing, we isolated two cDNA sequences with the longest ORF of 1410 and 1236 bp in length, which corresponds to predicted proteins with 469 and 413 amino acids, respectively. By alignment with other insect USP sequences, it is revealed that the deduced amino acid sequence of these two sequences encoded homologs of CsUSP1 and CsUSP2 and are predicted to be unstable proteins with calculated molecular masses of 52.65 and 46.79 KDa, respectively.

### Structural Analysis of CsEcRs and CsUSPs

**FIGURE 1:**
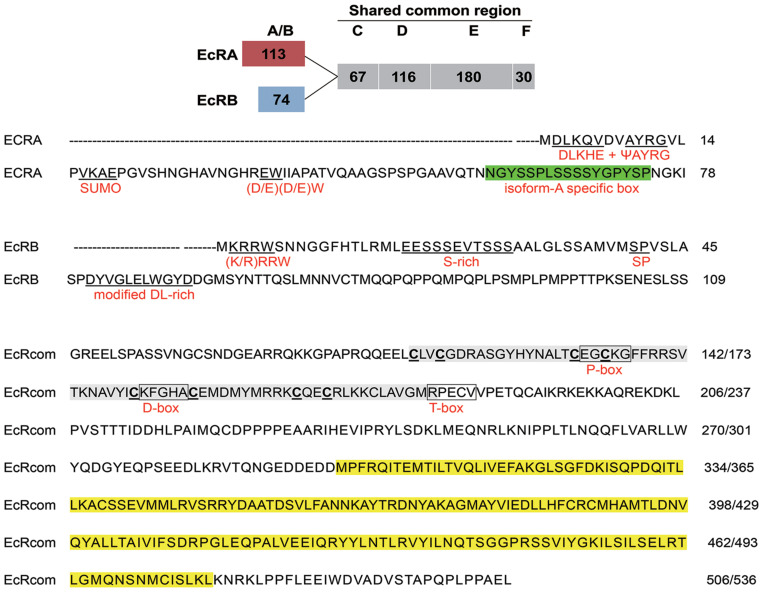
Schematic diagram of CsEcRA and CsEcRB structure (top) and sequence of two CsEcR isoforms with putative motifs (bottom). The numbers of amino acids in each domain are presented in frame, including A/B domain (N-terminal domain for independent transcriptional activation; C domain (DNA-binding domain, DBD); D domain (hige region); E domain (ligand-binding domain, LBD); F domain (C-terminal region). Conserved motifs in CsEcRA include a Type 2 isoform-A specific box, two conserved N-terminal sequences (DLKHE and ΨAYRG), SUMOylation motif, and (D/E) (D/E) W residues. Conserved motifs in CsEcRB include a Type 6 isoform-B1 specific box, (K/R) RRW motif, S-rich motif, SP residues, and a modified DL-rich motif. Regions corresponding to C and E domains are highlighted in gray and yellow. Three conserved 5-aa motifs (P-box, D-box and T-box) are boxed. The cysteine residues of the zinc finger motifs in the C domain are outlined.

**FIGURE 2:**
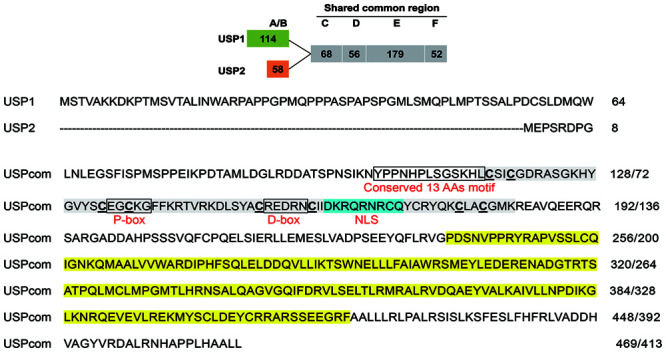
Schematic diagram of CsUSP1 and CsUSP2 structure (top) and sequence of two CsUSP isoforms with putative motifs (bottom). The numbers of amino acids in each domain are presented in frame, including A/B domain (N-terminal domain for independent transcriptional activation; C domain (DNA-binding domain, DBD); D domain (hige region); E domain (ligand-binding domain, LBD); F domain (C-terminal region). Regions corresponding to C and E domains are highlighted in gray and yellow. The putative P-box, D-box, and 13 amino acids motif conserved in all RXR/USPs located upstream from the C domain are boxed. The putative nuclear localization signal (NLS) is highlighted in blue.

CsEcRA and CsEcRB contain a common C-terminal region with DNA-binding (C domain) and ligand-binding domains (E domain) but unique termini of 113 and 74 amino acids (Figure 1). The A/B domain (N-terminal domain for independent transcriptional activation) of CsEcRA contains two conserved N-terminal sequences (DLKHE and ΨAYRG, where Ψ represents a large hydrophobic amino acid), one conserved microdomain (SUMOylation motif, small ubiquitin-related modifier ligases), and conserved (D/E) (D/E)W residues. Additionally, a modified Type 2 isoform-A-specific box (NGYSSPLSSSSYGPYSP) was identified in the C-terminal region of CsEcRA A/B domain. As with other reported lepidopteran insect EcRBs, the CsEcRB has a typical Type 6 isoform-B1-specific box which contains four microdomains [i.e., the (K/R)RRW motif, the S-rich motif (EESSSEVTSSS), the SP residues, and the highly modified DL-rich motif (D/E)Yx(E/D)LWxD]. In the shared common region of CsEcRA and CsEcRB, the cysteine residues of two zinc finger motifs are located in the C domain. The putative P-box and D-box are also located within the C domain, closely followed by the conserved T-box. Moreover, both CsEcRA and CsEcRB lack the nuclear localization signal (NLS) sequence. Similar to structures of CsEcRs, CsUSP1, and CsUSP2 contain a specific A/B domain (114 and 58, respectively) and a shared common region with both C (DNA-binding) and E (ligand-binding) domains (Figure 2). The 13 amino acid motif conserved in all USPs is located upstream from the C domain. The putative P-box and D-box are located in the C domain of CsUSP1 and CsUSP2, and the NLS sequence KRTVRK is located downstream of the two putative P-box and D-box motifs.

**FIGURE 3:**
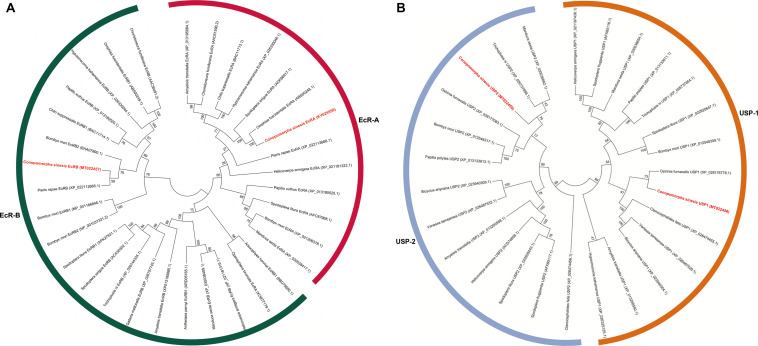
Phylogenetic analysis of lepidopteran ecdysone receptors **(A)** and ultraspiracle Proteins **(B)**. The phylogenetic tree was constructed by using the sequences of different lepidoptera families by a neighbor-joining (NJ) method with bootstrap of 1,000 replicates. *C. sinensis* sequence was used as outlier, and figure shows family wise grouping the lepidopteran EcRs and USPs.

The deduced CsEcRs and CsUSPs were compared with all other lepidopteran EcR and USP orthologs available from database NCBI. The evolutionary relationship of 13 EcRA, 20 EcRB, 12 USP1, and 13 USP2 derived from lepidopteran insects were evaluated after sequence alignment and phylogenetic analysis. In both EcR and USP phylogenetic trees, two isoforms were clustered in two separate clades by an NJ tree with high bootstrap supports. The results showed that CsEcRA and CsEcRB have the highest similarities to the EcRA from *Omphisa fuscidentalis* (Lepidoptera: Crambidae) and EcRB from *Pieris rapae* (Lepidoptera: Pieridae), respectively (Figure 3A). CsUSP1 was most closely resembles USP1 from *Ostrinia furnacalis* (Lepidoptera: Noctuidae) and CsUSP2 were most related to USP2 from *Trichoplusia ni* (Lepidoptera: Noctuidae) (Figure 3B).

### Developmental Expression Patterns of *CsEcR* and *CsUSP* Genes

**FIGURE 4:**
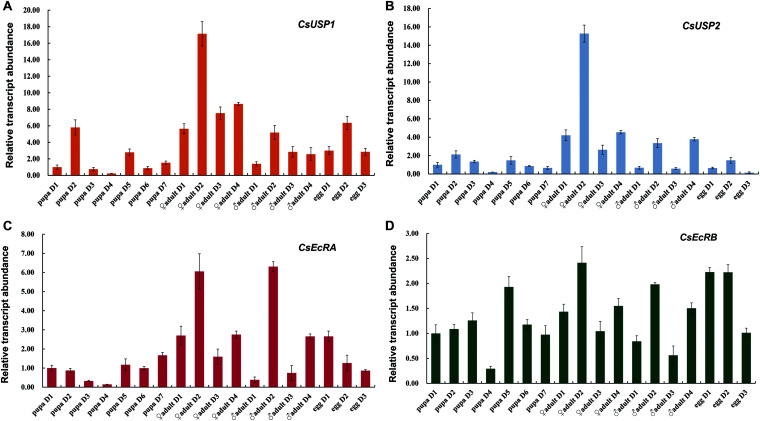
Developmental expression patterns of *CsEcR* and *CsUSP* genes in *C. sinensis*. Gene expression levels of CsUSP1 **(A)**, CsUSP2 **(B)**, CsEcRA **(C)**, and CsEcRB **(D)** at each time point were divided by that of the 1-day-old pupae. Each histogram bar represents mean ± SE of three independent replications with 10 pupae, 15 adults, or 200 eggs in each replicate. The β-*actin* expression levels were used as the internal control. The relative gene expression levels were calculated according to 2^–ΔΔCt^ method.

The qRT-PCR analysis with total RNA extracted from the whole bodies of pupae, adults males, adults females, and eggs revealed that Changes in mRNA expression levels of *CsEcRA*, *CsEcRB*, *CsUSP1*, and *CsUSP2* were different from each other during development in *C. sinensis* (Figure 4). *CsUSP1* showed a developmental expression profile different from that of *CsUSP2.* The transcription levels of *CsUSP1* were relatively low in pupae (except in 2-day-old pupa), started to increase after adult emergence, reached their peaks in 2-day-old adults, and stayed high in egg stage (Figure 4A). The transcription levels of *CsUSP2* were relatively high in female adults, followed by male adults, but were low in pupae and egg stages (Figure 4B). In addition, the expression levels of *CsUSP1* and *CsUSP2* in 2-day-old female adults were almost 3- and 5-folds higher than that of male adults. The expression profiles of *CsEcRA* and *CsEcRB* are significantly different (Figures 4C,D). The expression of *CsEcRA* was activated in 2-day-old adults and 1-day-old eggs, whereas small changes in the expression levels of *CsEcRB* were observed in different developmental stages. The transcription levels of *CsEcRA* were the highest in adult, followed by egg stage, but the lowest in pupa. Unlike *CsEcRA*, the expression levels of *CsEcRB* were steady in different developmental stages. Moreover, expression levels of the four tested genes were varied among different tissues of 4-day-old male and female adults (Supplementary Figure S1). These results suggested that *CsEcRA*, *CsEcRB*, *CsUSP1*, and *CsUSP2* play important but different roles in development of *C. sinensis*.

### Effect of Insecticides on *C. sinensis* Eggs

Bioassay results showed that insecticide toxicity on *C. sinensis* eggs was highly dependent on the insecticide used and the time of insecticide application. The susceptibility of *C. sinensis* eggs to chlorpyrifos was the highest among all four tested insecticides, with LC_50_ values for 1-, 2-, and 3-day-old eggs of 2.21, 9.07, and 25.35 mg/L, respectively. Chlorbenzuron and λ-cyhalothrin were moderately harmful to *C. sinensis* eggs with LC_50_ values for 1-day-old eggs of 4.01 and 4.66 mg/L, respectively. Ebufenozide had relatively low toxicity to *C. sinensis* eggs, with LC_50_ values more than 2–15 times higher than those of the other three insecticides. Interestingly, the LC_50_ values of the tested insecticides for 1-day-old eggs were lower than those of 2- or 3-day-old eggs. This was especially evident for eggs exposed to chlorbenzuron and chlorpyrifos; the LC_50_ values of chlorpyrifos and chlorbenzuron were 4- and 12-fold higher, respectively, for 3-day-old eggs than for 1-day-old eggs (Table 3).

**TABLE 3:** LC_50_ values (with corresponding 95% confidence intervals) for *C. sinensis* eggs after exposure to insecticides.

**Insecticides**	**Days of eggs**	**Regression equations**	***X*^2^(*df*)**	**LC_50_ (mg/L)**	**CI**	**No. treated**
Chlorpyrifos	D1	y = 4.21 + 2.31x	1.17 (3)	2.21	1.80-2.72	600^a^
	D2	y = 3.65 + 1.41x	3.97 (3)	9.07	5.97-13.76	600^a^
	D3	y = 2.82 + 1.55x	3.43 (3)	25.35	15.70-40.92	600^a^
Chlorbenzuron	D1	y = 4.32 + 1.12x	2.23 (3)	4.01	2.63-6.10	600^a^
	D2	y = 3.84 + 0.98x	4.96 (3)	15.36	7.18-32.88	600^a^
	D3	y = 3.58 + 0.96x	4.81 (3)	31.05	18.26-52.79	600^a^
λ-cyhalothrin	D1	y = 4.32 + 1.02x	2.37 (3)	4.66	2.85-7.62	600^a^
	D2	y = 3.81 + 1.04x	3.58 (3)	14.11	8.81-22.58	600^a^
	D3	y = 3.56 + 1.01x	4.11 (3)	26.94	16.83-43.13	600^a^
Tebufenozide	D1	y = 3.28 + 1.13x	5.24 (3)	33.14	20.66-53.17	600^a^
	D2	y = 2.86 + 1.31x	7.63 (3)	42.90	26.33-69.91	600^a^
	D3	y = 2.82 + 1.35x	6.12 (3)	40.54	25.62-64.15	600^a^

**The results are presented as regression equations, degree of freedom (df), LC_50_, corresponding 95% confidence intervals (CI), number of treated embryos. Low X^2^ value (<11.00) indicates the data adequacy to the probit model used to estimate the mortality curves. ^*a*^40 eggs per replicate, 3 replicates per dose, 5 doses per assay.**

**FIGURE 5:**
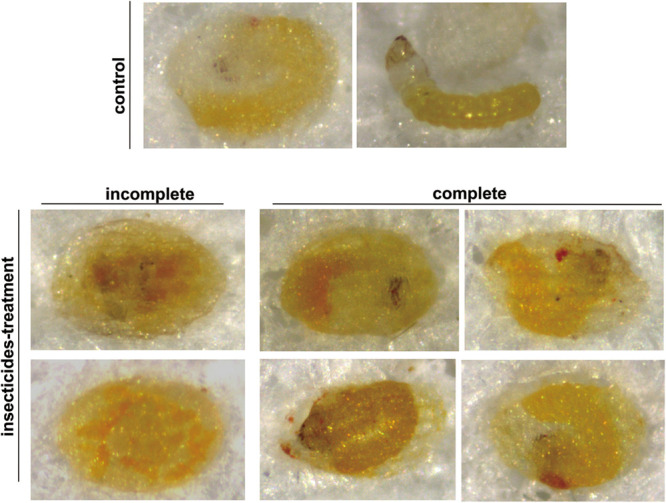
Lethal phenotypes caused by insecticides exposure in *C. sinensis* eggs. Normal embryonic development observed in control group **(top)** and lethal phenotypes of *C. sinensis* eggs with incomplete or complete embryonic development caused by insecticides **(bottom)**.

The lethal phenotypes caused by sublethal concentrations of chlorpyrifos, chlorbenzuron, and λ-cyhalothrin were similar, although the mechanisms of action were quite different. A small number of abnormal *C. sinensis* eggs in treatment groups died during embryonic development (incomplete), whereas a large number of abnormal eggs were observed to have apparently fully developed inside the eggshell but died at the moment that normal eclosion would occur (complete) (Figure 5). Additionally, the most typical morphological defects of insecticide-treated *C. sinensis* eggs (i.e., eggs which are complete developed but die inside the eggshell) were microscopically examined after dissection (Supplementary Figure S2). Most of the unhatched larvae inside the eggshells were spongy, twisted, and small compared with the control group.

### Embryonic Response of *C. sinensis* Genes After Sublethal Concentration Insecticide Exposure

**FIGURE 6:**
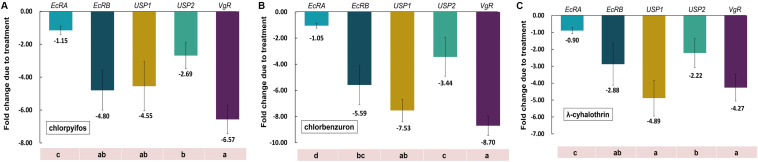
Effects of selected insecticides on the gene expression of *CsEcR*s, *CsUSP*s and *CsVgR* in *C. sinensis*. The figure shows the gene expression of *CsEcRA*, *CsEcRB*, *CsUSP1*, *CsUSP2*, and *CsVgR* in *C. sinensis* after different treatments with chlorpyrifos **(A)**, chlorbenzuron **(B)**, and l-cyhalothrin **(C)**. The bars represent the mean ± SE of the relative gene expression of three independent replications with β-*actin* as the housekeeping gene. The fold changes due to treatments were calculated according to the comparative Ct method. Different lowercase letters above the columns indicate significant differences (Tukey’s test, *P* < 0.05).

To address the impact of sublethal concentrations of selected insecticides on gene expression of *CsEcR*s, *CsUSP*s, and *CsVgR* in *C. sinensis* eggs, the relative mRNA expression of these genes was determined using qRT-PCR. The results revealed that all the tested genes had variable responses to different insecticide treatments (Figure 6). The five tested genes shared similar variation trend after exposure to tested insecticides. Among them, *CsVgR* and *CsUSP1* were the most sensitive to all the tested insecticides, and the change of transcriptional abundance was diminished 4.55–7.53-fold and 4.27–8.70-fold, respectively, compared with the control group. The mRNA levels of *CsEcRB* and *CsUSP2* were reduced 2.88–5.59-fold and 2.22–3.44-fold, respectively, after insecticide exposure. Intriguingly, no adverse impact on transcription level of *CsEcRA* was observed in chlorpyrifos-, chlorbenzuron-, or λ-cyhalothrin-treated groups.

## Discussion

### Analysis of CsEcRs and CsUSPs

In this study, we cloned and sequenced the genes for two EcR isoforms, CsEcRA and CsEcRB as well as the heterodimer partners, CsUSP1 and CsUSP2, using a cDNA library constructed from *C. sinensis* eggs. The four dimerizing partners of the functional ecdysone receptor, EcR and USP genes, were identified the first time from the Gracillariidae insects. Two USP isoforms have already been described in *Manduca sexta* (Lepidoptera: Bomycoidae) (Jindra et al., 1996), *B. mori* (Cheng et al., 2008), and *Spodoptera frugiperda* (Lepidoptera: Noctuidae) (Giraudo et al., 2013) and allowed us to identify the two USP isoforms in *C. sinensis.* Conversely, there are three EcR isoforms (A, B1, and B2) reported in *D. melanogaster* and *Daphnia magna* (Crustacea: Cladocera), whereas there are two isoforms of EcR (A and B) reported in all sequenced lepidopteron species (Jindra et al., 1996; Minakuchi et al., 2003; Yao et al., 2010). These EcR and USP variants are produced by alternative splicing and their expression is regulated by distinct promoters in insects (Koelle et al., 1991; Schwedes and Carney, 2012). Despite the transcriptome study results for *C. sinensis* adults previously reported by our research group, we could not rule out the possible existence of a third isoform of EcR in *C. sinensis* due to the lack of genetic information in larvae and pupae.

CsEcRA and CsEcRB are two different proteins with distinct N-terminal A/B domain with sequences of 113 and 74 amino acid residues, respectively, and share a common C-terminus of 393 amino acids. Similar to CsEcRA and CsEcRB, the amino acid sequence alignment indicated that CsUSP1 and CsUSP2 include five typical domains normally present in the superfamily members of nuclear receptors: ligand-independent activation A/B domain, a two-zinc-finger DNA-binding domains (C domain), a hinge region (D domain), a ligand-binding domain (E domain), and a poorly conserved carboxyterminal region (F domain).

The two critical functional domains, the C domain and the E domain, are highly conserved across arthropods, and *C. sinensis* is not an exception (Evans, 1988; Schwedes and Carney, 2012). In the region of the C domain, there are two zinc-finger motifs containing a proximal P-box and D-box sequences. The putative P-box and D-box are both 5-aa motifs and are involved in specific half-site recognition and half-site spacing recognition. The P-box (EGCKG) of CsEcRs and CsUSPs is 100% identical to the EcR/USP of other arthropod species, whereas the D-box is less conserved across arthropods (Umesono and Evans, 1989; Bortolin et al., 2011).

The T-box in the shared common region of EcR isoforms is extremely conserved across arthropods, closely followed by the zinc-finger motifs. Substitutions in sequence of the putative motifs could point to the possibility of differences in isoform-specific interactions with ligands and DNA-target sequences (White et al., 1997; Tzertzinis et al., 2010). The E domain plays a critical role in ligand binding and is highly conserved across arthropods, since the hormone 20E is the acknowledged molting hormone in this group. The high similarity of CsEcRs and CsUSPs to the orthologs of all other arthropods indicated that ecdysone is also the molting hormone in *C. sinensis*.

In CsUSPs, the nuclear localization signal (NLS) sequence KRTVRK, which has been identified in regions with rich basic amino acids arginine and lysine, locates right after the two putative P- and D-box regions in *C. sinensis*. The position of the NLS varies among insect species. NLS was found to be located in the C domain of USPs in *D. melanogaster* but in the D domain of USP from *Plutella xylostella* (L.) (Lepidoptera: Plutellidae) (Tang et al., 2012). Moreover, the NLS of EcR isoforms is conserved among all species except for Heteroptera and Lepidoptera, reflecting functional differences in the anchoring of these proteins to transporter proteins in Heteroptera and Lepidoptera (Boulikas, 1993; Watanabe et al., 2010).

### Susceptibility of *C. sinensis* Eggs to Insecticides

The susceptibility of insect eggs to insecticides varies widely and is both chemically and temporally dependent. Exposure to different insecticides showed no negative impact on viability of eggs in *Harpalus pennsylvanicus* (Coleoptera: Carabidae), *Triatoma infestans* (Hemiptera: Reduviidae), or *Chrysoperla carnea* (Neuroptera: Chrysopidae) (Kunkel et al., 2001; Toloza et al., 2008; Amarasekare and Shearer, 2013). In contrast, some insecticides had varying degrees of toxicity to the eggs of *Lobesia botrana* (Lepidoptera: Tortricidae), *Orius insidiosus* (Hemiptera: Anthocoridae), *Ceraeochrysa cubana* (Neuroptera: Chrysopidae), *Cimex lectularius* (Hemiptera: Cimicidae), and *Tamarixia triozae* (Hymenoptera: Eulophidae) (Ioriatti et al., 2009; Gontijo et al., 2015; Rugno et al., 2015; Hinson et al., 2016; Morales et al., 2018).

Egg susceptibilities to insecticides are considered a vitally important factor for control of insect pests, especially of borer insects and leaf miners whose larval and adult life stages find refuge from insecticidal exposure within plant hosts. Some success with respect of ovicidal activity using single or combination agents has been reported in different borer and leaf miner insect species. Ninety five percent reduction of egg viability was observed in *Diatraea grandiosella* (Lepidoptera: Grambidae) eggs with a combination of 100 or 200 mg/L tebufenozide and methoxyfenozide (Trisyono and Chippendale, 1998). The egg hatchability of citrus peelminer, *Marmara gulosa* (Lepidoptera: Gracillariidae), was reduced to between 0.75 and 41.25% after exposure to various insecticide treatments (Grafton-Cardwell et al., 2008). Methomyl and cartap hydrochloride exhibited high toxicity and caused mortality more than 80% of eggs of *Neoleucinodes elegantalis* (Lepidoptera: Crambidae) (Silva et al., 2018). In contrast, the hatchability of insecticide-treated eggs was not adversely affected in tomato leaf miner *Tuta absoluta* (Lepidoptera: Gelechiidae).

Two moderately toxic insecticides (chlorpyrifos and λ-cyhalothrin) and one insect growth regulator (chlorbenzuron) showed considerable toxicity to *C. sinensis* eggs in this study. The ovicidal effect of these insecticides was confirmed by morphological observation of defects. The data indicated the potential utility of such compounds as ovicides and provides new approaches to chemical control of *C. sinensis*. However, lower ovicidal activity was observed when 2- or 3-day-old eggs of *C. sinensis* were exposed to the tested insecticides, indicating lower susceptibility of older eggs. This might be the explanation for the lack of efficacy observed in field trials.

### Embryonic Response of *CsEcRs* and *CsUSPs* to Insecticides

As a controversial issue, insecticide use is at the regulatory forefront in most countries (Smagghe et al., 2019). Efficacy assessments of insecticide application usually focus on the mortality of arthropod pest species. Comprehensive risk assessments need to be conducted on the biological, molecular, and biochemical responses of target species and other ecological factors for rational use of insecticides to improve human and environmental safety. Insecticides may affect numerous physiological processes by interacting with primary and secondary sites of action within an individual organism and directly or indirectly lead to lethal or sublethal consequences that compromise its homeostasis, survival, and reproduction (Guedes et al., 2016). Disturbing the ecdysone signal pathway is among the toxic responses to topical, residual, or dietary exposure to insecticides. The alterations of normal hormonal signaling pathways by environmentally harmful agents can lead to adverse effects on insects at both the individual and population levels (Gaertner et al., 2012; Guedes et al., 2016). Additionally, a cascade of changes in the expression of regulatory factors is sequentially upregulated or downregulated by those agents in different developmental stages of insects.

Due to the insufficient data on ecdysone receptor identification and characterization in *C. sinensis*, there is no information about the ecdysone receptors transcriptional level in *C. sinensis* and no studies which have evaluated the role of ecdysone receptors in response to insecticide exposure. Hence, we obtain and characterize sequence information of EcR and USP from *C. sinensis*, and investigated their embryonic expression alteration after exposure to three common used insecticides in *C. sinensis* control. *CsVgR* was also selected for expression detection based on our previous study about reproductive responses to insecticides in *C. sinensis* adults. Differential impact on the expression of key endocrine- and reproductive-related genes was observed in insecticide-treated *C. sinensis* eggs. Drastic reduction of transcript abundance of *CsUSP1* and *CsVgR* (4.55–7.53 and 4.27–8.70 times, respectively), followed by a lesser decrease of *CsUSP2* and *CsEcRB* expression (2.88–5.59 times and 2.22–3.44 times, respectively), was observed after exposure to chloripyifos, chlorbenzuron, and λ-cyhalothrin, whereas no variations of *CsEcRA* expression were observed in insecticide-treated *C. sinensis* eggs. In other research reports, long-term exposure to ecdysone and a juvenile hormone agonist was previously found to increase the expression of *EcR* and *USP* in *Cydia pomonella* linnaeus (Lepidoptera: Tortricidae) adults, *Bombyx mori* (Lepidoptera: Bombycidae) larvae, and *Spodoptera frugiperda* (Lepidoptera: Noctuidae) larvae (Sun et al., 2003; Parlak, 2012; Giraudo et al., 2013). A significant increase in *EcR* transcriptional expression was observed after exposure to a highly toxic insecticide (fipronil) for 30 h in *Amphiascus tenuiremis* (Copepoda: Harpacticoida) adults (Gaertner et al., 2012). Sulfathiazole exposure for 12 h induced a 2-fold change of transcriptional abundance of *EcR* and *USP* in *Chironomus riparius* (Diptera: Chironomidae) larvae (Park and Kwak, 2018). These studies suggest that the differential impact of insecticides on expression of *EcR* and *USP* are due in part to the developmental stage of target insect and exposure duration of treatments.

In summary, the results presented here on cDNAs encoding ecdysone receptor proteins from a gracillariidaes insect and the transcript variation observed after insecticide exposure provide a more comprehensive understanding of these genes in terms of insecticide-responsive gene expression. Owing to the rapid and more drastic reduction in the transcriptional abundance, *CsVgR* and *CsUSP1* might be two appropriately sensitive biomarkers for ovicidal effect assessment of insecticides in *C. sinensis*. Furthermore, similar variation trends of the five tested genes and similar morphological abnormalities were observed after exposure to different insecticides in *C. sinensis* eggs. The results suggested that the ovicidal effects of different insecticides might be based not only on differences in mechanism of action but also on unidentified *in vivo* parameters, such as permeability of the eggshell, uptake/excretion, or metabolic detoxification of individual organism. However, more solid evidence regarding developmental stage-specific differential responses in metabolism or detoxification of target pests is required to substantiate this hypothesis.

## Data Availability Statement

The datasets presented in this study can be found in online repositories. The names of the repository/repositories and accession number(s) can be found in the article/Supplementary Material.

## Author Contributions

QY conceived the study, conducted the experiments, and drafted the preliminary manuscript. QY, LQ, SX, and YD interpreted the results. BC refined and approved the final manuscript. All authors contributed to the article and approved the submitted version.

## Conflict of Interest

The authors declare that the research was conducted in the absence of any commercial or financial relationships that could be construed as a potential conflict of interest.

## Footnotes

1 https://www.ncbi.nlm.nih.gov/orffinder/

2 http://expasy.org/tools/dna.html

3 https://web.expasy.org/protparam/

4 http://www.cbs.dtu.dk/services/TMHMM/

5 http://www.cbs.dtu.dk/services/SignalP/

6 http://psort.hgc.jp/form2.html

7 http://www.ncbi.nlm.nih.gov/Structure/cdd/wrpsb.cgi

8 http://www.ebi.ac.uk/clustalw/index.html
