# Supplementary material for: Ecdysone Receptor (EcR) and Ultraspiracle Protein (USP) Genes From Conopomorpha sinensis Bradley Eggs: Identification and Expression in Response to Insecticides
Source: Front Physiol. 2020 Jul 17;11:851. doi: 10.3389/fphys.2020.00851 (PMC7380065; doi:10.3389/fphys.2020.00851)
Supplement: Supplementary file 1 [file Table_1.DOCX]

**Supplementary table S1.** The concentration range of the tested insecticides.

|  | 1-day-old (mg.a.i./liter) | 2-days-old (mg.a.i./liter) | 3-days-old (mg.a.i./liter) |
| --- | --- | --- | --- |
| chlorpyrifos | 0.625-10 | 1-16 | 2-32 |
| chlorbenzuron | 0.625-10 | 1-16 | 4-64 |
| [λ-cyhalothrin](javascript:;) | 0.625-10 | 2-32 | 4-64 |
| tebufenozide | 0.625-10 | 4-64 | 4-64 |
