# Supplementary figures and images for: Ecdysone Receptor (EcR) and Ultraspiracle Protein (USP) Genes From Conopomorpha sinensis Bradley Eggs: Identification and Expression in Response to Insecticides
Source: Front Physiol. 2020 Jul 17;11:851. doi: 10.3389/fphys.2020.00851 (PMC7380065; doi:10.3389/fphys.2020.00851)

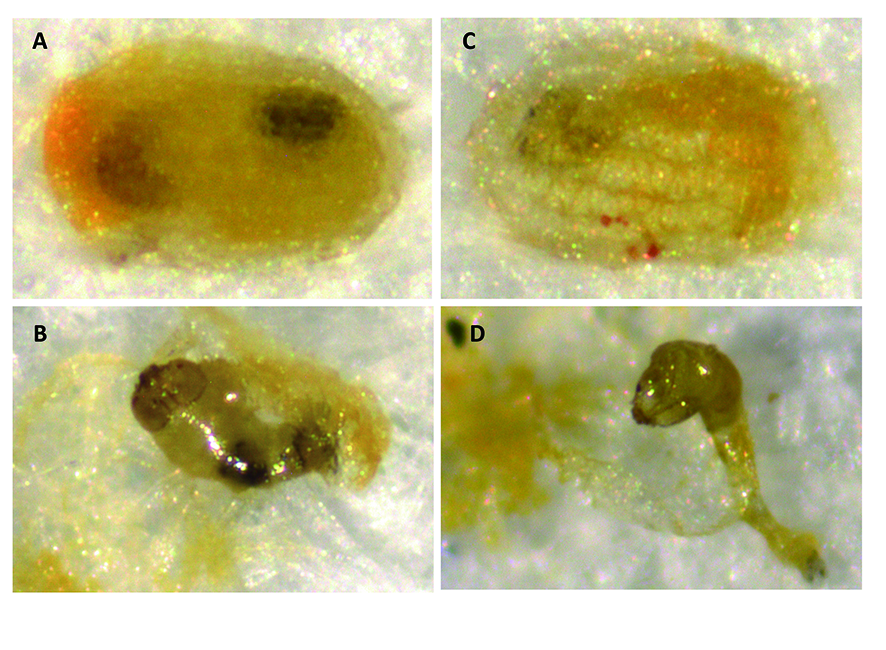

Supplement: Supplementary file 2 [file Image_1.TIF]
